# Learning and Collaboration during Crisis: A Novel University-Community Partnership to Manufacture Medical Personal Protective Equipment

**DOI:** 10.3390/ijerph18052258

**Published:** 2021-02-25

**Authors:** Alexandra H. Vinson, Astrid B. Fishstrom, Deborah M. Rooney

**Affiliations:** Department of Learning Health Sciences, University of Michigan, Ann Arbor, MI 48109, USA; astridf@med.umich.edu (A.B.F.); dmrooney@med.umich.edu (D.M.R.)

**Keywords:** organizational learning, disaster response, COVID-19

## Abstract

Research on crisis management focuses on pre-planning for disasters in order to understand potential barriers. However, one significant barrier to crisis response is that organizations may come together in unplanned configurations during crisis response. This means that significant opportunities exist for understanding the process by which individuals learn, collaborate, and create new systems during crises. In this case report, we present the case of face shield production by a university, academic medical center, and community partners during the supply chain collapse of the early COVID-19 pandemic with the aim of identifying the relationships that formed during the COVID-19 response, so that this case of relationship formation and participant experiences might inform similar disaster response challenges in the future. Thirteen participants responded to an in-depth questionnaire designed to simulate an asynchronous in-depth interview. Respondents reported on the activities of 80 individuals from 38 units/organizations, providing insight into communication challenges and resolutions. Responses were analyzed using thematic analysis, highlighting roles and relationships among participants. The findings grant insight into the experience of learning from crisis response efforts, responding to recent calls for social scientific work on COVID-19 responses.

## 1. Introduction

Past research on crisis management has focused on the role of organizational pre-planning for disasters [1]. Much of this research has focused on understanding past crises in order to wisely plan for future ones, and scholars have found that a retrospective evaluation can identify barriers to practical disaster response [2]. One such barrier is role confusion, including unclear reporting and management structures among those who respond to disasters, as shown by one study of community health workers [3]. Another barrier is that idealized configurations of disaster response organizations can differ greatly from the actual relationships that form between organizations when they respond to a real disaster [4]. This may particularly be the case when organizations collaborate across sectors, such as government-university-industry partnerships, since these organizations have salient differences in culture and operational constraints [5]. For all these reasons, how response events play out during a critical event may differ greatly from a planned response, supporting the role of learning from past disasters. Thus, significant opportunities exist for understanding the process by which individuals learn, collaborate, and create new systems during crises.

When difficulties in inter-agency coordination and communication have been studied, researchers have identified the role of shared mental models in the decision-making process, such that coordinating participants’ mental models of how to respond must take place before the actual response can proceed smoothly [6]. These studies make a strong case for looking at how individuals participate in disaster responses, understanding individuals as members of response organizations who actively interpret organizational standards and guidelines in their responses. Research in this vein provides an important warrant for shifting attention to task and knowledge sharing and coordination [7], which can be understood as aspects of the learning process.

These past studies have tended to focus on existing organizations that may come together in novel coalitions during a disaster. However, they focus less frequently on organizations that take up a new task in addition to coordinating in new ways with other organizations. As we face more novel disaster scenarios, as in the case of the coronavirus pandemic, understanding both the response patterns of established organizations, as well as the emergence and coordination of new disaster responses, is crucial. One way to study new organizational responses to disasters is to draw on qualitative social science approaches. Recently, there has been a call to bring more social scientific perspectives to the understanding of COVID-19 management [8]. Therefore, one objective of our case report is to use qualitative social scientific approaches to describe a case of COVID-19 response.

In this case report, we discuss a case of organizational coordination that took place as producers, designers, and procurers across an academic medical system, a university, and a local community identified each other and began to work together to produce personal protective equipment for healthcare workers. We draw on qualitative social science perspectives and methods to gain insight into the experiential aspects of participation in disaster response efforts. While disaster responses may differ from the plans that were designed to guide them (as highlighted by Chen, Zhang, Tadikmalla et al., 2019) [4], some crises strike organizations that did not have specific response protocols for the particular disaster at hand. In our case, the collapsing supply chains in the early weeks of the COVID-19 pandemic brought university and community members together in unexpected configurations to solve the challenging problem of producing personal protective equipment for the local health system. We present this case of rapid network formation, focusing on the experiences of key participants, including their communication practices and roles, to demonstrate the experiential aspects of learning during disaster response.

## 2. Materials and Methods

### 2.1. Case Description

On 10 March 2020, over 700 coronavirus (SARS-CoV-2 or COVID-19) cases were confirmed in the United States [9]. US healthcare facilities and regional hospitals, including the health system at the academic medical center described in this case, prepared for an anticipated flood of patients presenting with coronavirus-related illnesses.

As the health system’s supply leadership team watched the state’s COVID-19 cases increase exponentially [10], they recognized the burden that the growing national demand for PPE would have on the local healthcare system. Discussions with distributors alerted the supply leadership team of anticipated manufacturers’ supply chain challenges. They quickly prioritized the acquisition of face shields, which were recognized as an acceptable mechanism to increase longevity of N95 masks [11]. At the time, the health system’s average daily use rate, also called “the burn rate,” was 80 face shields per day. Anticipating a 100-fold increase at their maximum bed-capacity, the supply leadership team considered alternative PPE sources, including those across the university campus.

Soon after, a concerted effort began to produce face shields by mobilizing existing laboratory and makerspaces at the university and in the local community. However, since this was an entirely new effort, it was not clear which university and community members had the expertise to produce face shields, which spaces had the capacity to produce face shields, and which face shield model would be the best one to produce.

### 2.2. Study Design

In order to learn about the process by which disparate university and community groups collaborated in the effort to produce face shields, we constructed an in-depth questionnaire composed of 18 free-response questions (Table 1). This approach was designed to simulate an asynchronous in-depth interview, allowing us to gather detailed information from participants and their many contacts, who were actively learning how to respond to the COVID-19 crisis in healthcare. We organized the questions into three domains: Background, Collaborators, and Personal Experience. For the purposes of this research, we define collaboration as working together on a shared initiative.

### 2.3. Data Collection

By administering an in-depth questionnaire, our aim was to enable respondents to answer the questions slowly and to consult their email correspondence and personal records for relevant details. Following Institutional Review Board determination of exempt approval, the in-depth questionnaire was sent to 31 individuals who were involved in the face shield response. Participants gave informed consent to respond to the questionnaire. A reminder was sent to those who did not respond, and the final response tally was 13, resulting in a response rate of 41.9%. Respondents provided contact information, but the responses are presented anonymously in this analysis. While this is a small sample, the respondents represented nearly all of the known groups involved in the face shield response efforts—and they were able to share information on their contacts and communication with others in the response. As discussed below, this allowed us to understand the roles and activities of a total of 80 individuals from 38 university units and community organizations. Further, other studies of disaster response have used samples of comparable size [6,12]. Combined with the qualitative nature of the Personal Experience data, which help us understand motivations, challenges, and other elements of lived experience, our small sample size provided us with sufficient understanding to conduct our thematic analysis [13].

### 2.4. Data Analysis

Responses to Personal Experience questions were analyzed using a thematic analysis process [14]. This method proceeds through iterative cycles of familiarizing oneself with the data, assigning codes to data excerpts, organizing codes to generate themes, and refining themes through continual engagement with the data. The data were coded inductively, using word processing and Microsoft Excel 2016 to categorize similar excerpts from the survey responses. By working in iterative cycles to categorize the data according to codes identified by the research team based on the content of the excerpts, the research team was able to generate themes. Example themes, which are discussed in greater detail below, included: identification and acquisition of resources, coordination of participants, and getting approvals for proposed face shield designs. All authors participated in data analysis, using a consensus-based process to reach agreement on the meaning of codes and development of themes. In addition to the thematic analysis of questionnaire responses, responses to Background and Collaborators questions were used to assess the reach of the face shield production community that formed during the COVID-19 response. The questions asked respondents to list everyone they had contact with during their participation in the face shield response efforts, and our 13 respondents named 80 individuals from 25 university units and 13 community organizations (Table 2; some units have been combined or had their names generalized to preserve respondent anonymity; community organizations are not listed). The university units were concentrated in the engineering school, the university’s library system, and the university’s academic medical center, which includes the medical school. The community organizations included industry partners who donated materials and community makerspaces with face shield manufacturing equipment.

## 3. Results

In our analysis, we focused on the *experience* of participants in face shield production efforts, trying to understand the challenges and surprises they encountered, as well as the solutions they identified, and the lessons learned as they discovered resources and likeminded collaborators. We define collaboration as the act of working together on a shared initiative or problem. In past research on collaboration, specifically on collaborative governance, researchers have theorized the components of collaboration, emphasizing behaviors such as “sharing, participation, cooperation, communication, and agreement based on compromises, trust, mutual understanding, and sharing of information and resources” ([15], p. 4). The researchers describe the importance of positive interactions between collaborators in the dynamic and uncertain environments that often necessitate new collaborations. Building on their insights, we use qualitative methods to share participants’ experiences with collaboration in their own words.

### 3.1. Network Formation and Coordination

As noted above, our 13 respondents identified 80 individuals from 25 university units and 13 community organizations who were involved in the face shield response efforts. These questionnaire responses allowed us to characterize respondents’ participation in the face shield response efforts and organize them into four groups, described in greater detail in this section. In what follows, we qualitatively describe the face shield response as a rapidly-forming network of individuals spanning university and community partners.

There were four primary participant types in the face shield response efforts: Connectors, Coordinators, Consultants, and Contributors.

*Connectors.* Individuals who introduced participants to each other, but were not otherwise significantly involved in the response efforts. Connectors were valuable in linking individuals across university and health system units, and between the university and community.

*Coordinators.* The main leaders of the response efforts who worked closely together to reach all of the participants, sharing information and coordinating production and delivery of face shields to the health system. We define coordination as a special role within a collaboration, wherein coordinators organize the activity of the shared initiative, in our case, the design, production, and donation of face shield personal protective equipment.

*Consultants.* Individuals employed primarily by the academic medical system who served in approval roles related to design specifications, safety, and disinfection protocols.

*Contributors.* University and community members who received design specifications from Coordinators, participated in production, donated or procured materials, and managed the collection of finished face shields from other Contributors. Yet other Contributors were university leaders who needed to be knowledgeable on the flow of university resources and faculty, staff, and student efforts, but who did not produce face shields themselves. Most response participants were Contributors.

Of all of the groups, Coordinators faced the highest learning burden and were important for reducing the learning burden of Contributors by providing clear instructions and developing functional protocols for producing, collecting, distributing, and cleaning the face shields. Coordinators’ learning tasks included discovering other network participants (via Connectors) and finding out what other network participants knew, figuring out which individuals were knowledgeable about medical system regulations (Consultants), identifying manufacturing equipment and volunteers at the university and in the community (Contributors), and connecting these Contributors through infrastructures such as an online portal and protocols for the production and delivery of face shields. Thus, in our findings, we observed a horizontal collaboration among groups of Contributors, as well as an emerging vertical approval chain within the health system administration. The Coordinators formed a pivot point, both helping to coordinate the activity of the Contributors, while working with Connectors to identify Consultants and identify the necessary approval chain.

In addition to various participant types, we also identified three distinct types of relationships between network members:

*New Relationships.* These relationships formed during the face shield response, often facilitated by Connectors and strengthened by longer-term engagement with Coordinators. Forming these relationships allowed the Connectors and Coordinators to identify the full scope of resources available within the university and community setting so that Coordinators could relay information to Consultants, helping the health system anticipate the supply and adjust supply chains.

*Established Relationships*. These included everyday relationships with colleagues that predated the COVID-19 pandemic. These relationships helped individuals compose teams within their units, relying on pre-existing institutional processes to carry out the novel task of designing, producing, approving, and collecting face shields. Many of the Consultants, who served in approval roles for design specifications, raw materials procurement, and collection of finished face shields, had pre-existing relationships with other Consultants. There were also longstanding relationships between the university and community makerspaces, who as Contributors were able to share resources openly, including providing component materials such as sheet plastic. In addition, the established relationships allowed for groups to share information and move quickly in an environment of trust.

*Established Weak Ties*. These relationships, which had a surprising value to our respondents, were pre-existing relationships characterized by a weak tie that was able to be drawn on in the new context of the face shield response. However, weak relationships developed more slowly as Coordinators gained confidence that, for example, community partners could fulfill their commitments quickly and that they were providing accurate information. The value of weak ties in this case evokes classic work in social science, whereby weak ties help individuals access new information that they would not otherwise get via their strong ties [16]. While we did not collect data that would enable a formal social network analysis, we draw on this terminology here to relate our findings to this long-studied property of social networks.

### 3.2. Thematic Analysis of Response Efforts

While the formation of the network and our descriptions of the types of participants and relationships that characterize this network are important for understanding the face shield response from an organizational perspective, those findings cannot shed light on the experience of participating in the response efforts. In order to study respondents’ individual experience, we analyzed the Personal Experience survey questions to identify themes related to challenges, solutions, surprises, and lessons learned that were identified by respondents. This thematic analysis generated 12 themes. In the following section, we discuss selected themes in greater detail.

#### 3.2.1. Challenges and Solutions

Working within an established organizational structure to accomplish a new task posed challenges for the university members who participated in the face shield response. As one respondent noted:

*The University is designed for individual and team investigation and prototyping, but not large-scale production. Collaboration and project management across the University for a production effort on this scale required incredible effort.*

Respondents actively pursued solutions to resolve the challenges they encountered during the face shield production process. Here, we focus on three central challenges: identification and acquisition of resources, coordination of Contributors and Consultants, and getting approvals for the proposed face shield designs.

First, respondents faced challenges in coordinating the numerous individuals, university units, and community organizations involved in the face shield production process. The university was described by one respondent as a “distributed organization.” The university’s decentralized organizational structure made it difficult to find out who else was responding to the call to produce face shields, especially in the early days of the response. This attribute of the university heightened the importance of Connectors, who were able to identify and link individuals across disparate university units and within the local community. Additionally, the decentralized structure made it difficult for the Coordinators to manage various production teams, monitor progress, and keep track of design plans. As one respondent described:

*Multiple groups were launched without central coordination and numerous communications indicated that there were separate, overlapping and uncoordinated efforts trying to achieve a common end leading to substantial uncertainty and confusion.*

In response to these difficulties, Coordinators implemented a number of strategies, including a daily email update and frequent virtual meetings that allowed the Coordinators to share the latest information. Coordinators also relied on Connectors to find new Contributors and identify the proper Consultants needed to approve the face shield designs and distribution plans.

Second, participants faced challenges caused by the impact of COVID-19 on supply chains and logistics. COVID-19 affected supply chains across the United States and the world, which was difficult for the participants we surveyed, who had to create new supply chains in order to identify vendors and procure materials. Peréz-Mesa, Piedra-Muñoz, Galdeano-Gómez et al. [17] define a supply chain as a “series of operations,” highlighting the processual elements of producing and distributing goods, and how being attentive to supply chains as a process can help us understand how supply chains can be adjusted or created. Once the face shields had been produced, logistics challenges continued for the Coordinators and the Consultants: The face shields needed to be assembled, stored, and distributed, while avoiding cross-contamination among the multiple Contributor groups. Respondents reported several solutions, such as setting up a donation warehouse, working out transport plans, and building a web portal to connect the Coordinators, individuals responsible for the delivery of face shields to the health system, with the Contributors, individuals responsible for face shield production.

Finally, getting approvals was a prominent source of challenges among participants in the face shield response efforts and seemed to progress slowly. As approvals had to be gained for both the design and the materials before any production could commence, the pace of the response efforts was influenced by the timing of approvals. Some solutions discovered by the respondents included respecifying the approval chain, and there was a general sense that resolving the approvals challenges was a matter of communication.

#### 3.2.2. Surprises

As respondents navigated challenges and developed solutions, they encountered a number of surprises, including that informal networks were valuable, people were willing to share resources across unit and university/community boundaries, and there were many small businesses and people with 3-dimensional printers in the local community who wanted to help. Additionally, respondents were surprised at how valuable their pre-existing informal networks were in this process.

Two of the most important surprises encountered during the face shield production efforts were individuals’ willingness to collaborate and the “good humanity” that individuals demonstrated during the face shield response efforts. When describing willingness to collaborate, respondents described both drawing on established informal networks and coming together in novel collaborations:

*The value of informal networks and the willingness of people to share resources across school/college and [university]/community boundaries.*

*I thought it was awesome how so many groups got pulled together so quickly and, from my perspective, got working towards a common goal relatively quickly as well.*

Overall, we found that the need to produce face shields brought people together who might have otherwise never met, in the process forming novel infrastructures around a shared problem of interest.

#### 3.2.3. Lessons Learned

The final area in which respondents provided reflections on their experience was in their discussions of the lessons they learned during their participation in the face shield response efforts. One of the most prominent lessons learned was the value of informal networks and leaders who were “good connectors.” One respondent noted that leaders who were good connectors could “make the impossible possible.” During the face shield response, collaboration—working together on a shared problem—was a crucial component for effectively addressing the need, with respondents identifying the importance of bringing together the right people and prioritizing early coordination. While some respondents described drawing on existing connections as part of the face shield response, one respondent noted plans to retain contacts established during the face shield response in order to facilitate future work:

*I very much enjoyed being able to meet all of the collaborators I worked with, and appreciate their unique expertise in their areas of work. Feel like I could easily collaborate with them in [the] future.*

Responses similar to these suggest that the connections formed during this crisis response may reflect durable organizational learning and that it may be possible to reactivate these ties in the event of a future crisis.

## 4. Discussion

Past research on disaster response has demonstrated that collaborative effort, whether in a drill or real-life situation, can establish relationships and build trust [5]. These established relationships could then aid coordination in future disaster responses. Indeed, laying the groundwork for future collaborations is an important feature of both disaster preparation and disaster response [2,5,6], and disaster response frameworks are now being superseded by frameworks such as the Disaster Risk Management approach, which emphasizes prevention and readiness rather than reactivity [18]. By focusing on the individual experience of participation in a disaster response effort, our qualitative findings allow us to describe how relationships form during a disaster, focusing on their different types: Connector, Coordinator, Consultant, and Contributor. Moreover, our findings suggest that the connections formed during this crisis response may reflect durable organizational learning. This is important since it suggests that in the event of a COVID-19 resurgence or other disaster, it may be possible to reactivate these relationships, and move this constellation of collaborators from a response approach to a Disaster Risk Management [18] approach.

Indeed, our findings are important since past research on disaster response plans has pointed out that response plans may not include lessons learned from similar past events, introducing a risk that the same mistakes could be repeated in future epidemic responses [19], and reducing readiness and proactive preparation [18]. In addition, organizations may come together across sectors to support response efforts by lending the necessary expertise, such as in the case of academics advising the Puerto Rican government on how to plan the public health response to COVID-19 [20]. Such collaborations can create a division of labor for complex tasks that allows the collaborators to respond more effectively to the emergency at hand. These insights have led us to be attentive to lessons learned in these response efforts as we conducted our analysis. For example, respondents identified several lessons, including needing standards for communication, the need to spend more time planning before acting, and the importance of bringing together the right people and coordinating their efforts. We hope that by focusing on challenges, solutions, surprises, and lessons learned, and by sharing excerpts from these qualitative findings, future disaster planning efforts are able to draw on these insights.

## Figures and Tables

**Table 1:** Questionnaire administered to face shield response effort participants.

**Background**
1. What is your name, position, and affiliation?
2. On what date did you become aware of face shield shortages at the University?
3. How did you hear about the need for face shield production at the University?
4. Did you have direct contact with any COVID-19 response leadership members regarding face shield development?
5. When was your initial contact/correspondence with each of the COVID-19 response leader(s) you selected above?
**Collaborator(s)**
6. On what day did you begin working with collaborators on the face shield project?
7. Please explain the circumstances that led you to realize that collaboration would be necessary in order to move forward with your efforts.
8. Please describe your inclusion of collaborators from your own perspective.
9. If you worked with any lab mates/teammates on this project (students/trainees, staff, faculty, fellows, or other colleagues), please list them.
10. If you worked with any University affiliates outside of your lab/division, please list them along with their affiliation.
11. If you worked with any academic colleagues outside of the University, please list them, along with their institutional affiliation.
12. If you worked with any business/industry partners, please list them, being sure to specify the company name.
13. If you worked with any local community members, please list them, being sure to specify the organizational affiliation if appropriate.
14. Of all of the teammates and collaborators you listed above, had you worked with them previously? If so, please briefly describe your past interaction(s).
**Personal Experience**
15. What challenges did you encounter during the process of designing, developing, producing, and/or distributing face shield components?
16. Taking the problems you discussed above, how were each of these challenges resolved?
17. Was there anything that surprised you during your time working on this project?
18. Thinking back over the past few weeks, what are your biggest lessons learned from this project?

**Table 2:** Number of individuals by unit affiliation and role in the face shield response, as described by questionnaire respondents.

Role in Response	Unit	Number of Individuals
**Consultants**		**22**
	Health System COVID-19 Response Leaders	6
	Health and Safety/Infection Prevention	6
	Supply Chain	6
	Health System Donation Center	4
**Coordinators**		**4**
	Office of Technology Transfer	1
	Clinical Simulation Center/3D Innovation Lab	1
	Library Design Lab	1
	University Makerspace	1
**Connectors**		**4**
	Faculty and Leaders, School of Engineering	2
	Faculty, Medical School	1
	Student, College of Architecture and Urban Planning	1
**Contributors**		**50**
	University Makerspace	2
	College of Engineering	15
	College of Architecture and Urban Planning	4
	Medical School/Health System	3
	Clinical Simulation Center/3D Innovation Lab	8
	University Communications	3
	Other University Faculty	1
	Named Community Partners (individuals)	14
**TOTAL**		**80**

## Data Availability

The data are not publicly available due to the study design, participant consent, and/or IRB regulations.
